# Colorectal cancer: a qualitative study of coping strategies used by survivors, with associated social determinants

**DOI:** 10.1186/s13690-023-01104-4

**Published:** 2023-06-19

**Authors:** Antonio González-Herrera, Enriqueta Pujol-Ribera, Magdalena Esteva, Lorena Ruiz-Marcos, Sebastià March

**Affiliations:** 1 National School of Public Health (Carlos III Health Institute), Madrid, Spain; 2 Fundació Institut Universitari per a la recerca a l’Atenció Primària de Salut Jordi Gol i Gurina (IDIAPJGol), Barcelona, Spain; 3 Universitat Autònoma de Barcelona, Cerdanyola del Vallès, Spain; 4 Gerència Territorial de Barcelona, Institut Català de La Salut, Barcelona, Spain; 5 Primary Care Research Unit, Majorca Department of Primary Care, Baleares Health Service (IbSalut), Palma, Majorca Spain; 6 Health Research Institute of the Balearic Islands (IdISBa), Palma, Majorca Spain; 7 Faculty of Political Sciences and Sociology, Complutense University of Madrid, Pozuelo de Alarcón, Madrid, Spain; 8 Cooperativa Aplica, Madrid, Spain

**Keywords:** Colorectal neoplasms, Cancer survivors, Adaptation, Psychological, Social determinants of health, Psychology, Positive, Qualitative research

## Abstract

**Background:**

Colorectal cancer survivors have to develop coping strategies during the diagnosis and survivorship period. This study aims to identify coping strategies in patients with colorectal cancer, in particular the differences between coping strategies during the disease and throughout survival. It also aims to investigate the impact of some social determinants on coping strategies and critically reflect on the influence of *positive psychology*.

**Methods:**

Qualitative study with in-depth interviews of a purposive sample of 21 colorectal cancer survivors in Majorca (Spain), developed between 2017–2019. Data was analysed using interpretive thematic analysis.

**Results:**

We observed different coping strategies during the stages of disease and survival. However, striving toward acceptance and adaptation when facing difficulties and uncertainty, predominate in both stages. Confrontational attitudes are also considered important, as well as encouraging positive rather than negative feelings, which are considered unhelpful and to be avoided.

**Conclusions:**

Although coping during illness and survival can be classified into common categories (problem and emotion-centred strategies), the challenges of these stages are faced differently. Age, gender and the cultural influence of positive psychology strongly influence both stages and strategies.

## Background

Cancer is a leading cause of death globally. In 2020, cancer was responsible for 10,000,000 deaths [1], with colorectal cancer ranking second after lung cancer. In Spain, colorectal cancer is the first cause of death in women and the second leading cause of death in men [2]. In the last decades, cancer survival in Spain has increased and trends indicate that it will continue to increase. Specifically colorectal cancer survival has improved significantly [3].

Paradoxically, while many people have experienced the challenges of cancer diagnosis and treatment, our knowledge of their distress and the impact of the illness remain insufficient. Common issues in cancer survivors include sequelae of medical treatments, fears and concerns related to the whole management process, difficulties at work and in social relationships and changes in lifestyle and daily life [4]. In the specific case of rectal cancer, little is known about the emotional experiences of rectal cancer and the coping strategies used by rectal cancer survivors [5, 6].

In order to plan healthcare resources and to provide more empathetic assistance in accordance with their experience, it is crucial to understand how cancer patients cope with their disease. Psycho-oncology is a branch of oncology dedicated to shedding light on these experiences. Scientific literature is mostly quantitative and follows the Lazarus and Folkman model [7], which aims at classifying different profiles based on how patients evaluate the situation after the diagnosis, what strategies they deploy to face the problem and, finally, what are the results obtained throughout the process [8]. The Lazarus and Folkman model categorises coping strategies based on whether they focus on facing the problem or on managing emotions, and also on whether they use cognitive or behavioural strategies when looking for effective coping methods [9, 10].

Research on coping mechanisms of cancer survivors originates from the improved prognosis of many cancers [11] and most importantly, from the narrative of survivors revealing intense feelings of helplessness and uncertainty even when the disease is in remission [12]. It has been reported that these patients’ perception of their own health is similar to that of patients with severe chronic diseases [13]. In addition, it appears that the physical challenges, experiences and coping strategies change from the illness to the survival stage [14, 15].

There has been little research focused specifically on positive changes of this magnitude among cancer patients focused on cancer survival and coping strategies [15]. Our study, while inspired by the coping models used in quantitative research, uses qualitative methodology to deepen an understanding of the role of social determinants in these experiences. Chittem points out that qualitative research can help to explain the ways in which the patient chooses to deal with the cancer, and provides a background on the reasons and processes for this selection [16]. Qualitative research can yield information of great value as one of its main concerns is putting the individual’s experience as a priority [17].

This study aims to identify coping strategies in patients with colorectal cancer, in particular the differences between coping strategies during the disease and throughout survival. It also aims to explore the impact of some social factors on coping strategies and critically reflect on the influence of positive psychology.

## Methods

### Design, participants and sampling

A descriptive, qualitative study was conducted in the island of Majorca (Spain) between 2017 and 2019. We included patients diagnosed with colorectal cancer between 2011 and 2012 in the public hospitals in Majorca and identified through the Majorca cancer registry. Definition of survivor, met by all participants, was staying cancer-free 5 years after diagnosis and treatment. Patients with metastatic cancer, patients under active treatment and patients treated exclusively in private hospitals and those with cognitive impairment were excluded.

A sample of 21 survivors were included, after purposive selection using theoretical sampling aiming at discursive variability regarding age, sex, cancer location (colon/rectum), cancer stage and type of treatment received. Only one male patient with colon cancer, who agreed to participate, did not attend the appointment for the interview. Saturation was considered to be reached, and later interviews did not indicate any significant new concerns. Table 1 shows participants’ characteristics.

**Table 1:** Characteristics of colorectal cancer survivors interviewed. Majorca 2017–2019

**Code**	**Sex**	**Age**	**Civil status**	**Last occupation**	**Occupation type**	**Education**	**Cancer location**	**Stage**	**Colostomy**	**Chemo**	**Radio**
**E02_M46**	Man	46	Single	Salesman	Non manual	University	Colon	4	No	Yes	No
**E03_M54**	Man	54	Married	Driver	Manual	Secondary school	Colon	1	No	No	No
**E05_M61**	Man	61	Married	Builder	Manual	Primary school (not finished)	Rectum	3	No	Yes	Yes
**E06_M61**	Man	61	Married	Driver	Manual	Primary school	Colon	2	No	Yes	No
**E08_M65**	Man	65	Married	Waiter	Manual	Primary school	Rectum	2	Yes	Yes	Yes
**E10_M68**	Man	68	Married	Taxi driver	Manual	Primary school (not finished)	Rectum	2	Yes	Yes	No
**E11_M68**	Man	68	Married	Secondary school teacher	Non manual	University	Colon	2	No	No	No
**E13_M71**	Man	71	Married	Administrative officer	Non manual	University	Colon	2	No	No	No
**E15_M73**	Man	73	Married	Construction business owner	Non manual	Primary school	Colon	1	No	Yes	No
**E20_M80**	Man	80	Married	Restoration-builder	Manual	Primary school (not finished)	Rectum	2	Yes	Yes	Yes
**E21_M86**	Man	86	Widower	Company maintenance manager	Non manual	Secondary school	Colon	1	No	No	No
**E01_W41**	Woman	41	Divorced	Administrative officer	Non manual	Secondary school	Colon	3	No	Yes	No
**E04_W58**	Woman	58	Married	Housewife	Manual	NA	Colon	1	No	No	No
**E07_W61**	Woman	61	Married	Director’s pa	Non manual	Secondary school	Colon	3	No	Yes	No
**E09_W67**	Woman	67	Married	Housewife	Manual	Primary school (not finished)	Rectum	2	Yes	Yes	No
**E12_W68**	Woman	68	Married	Secretary	Non manual	Secondary school	Colon	3	No	Yes	No
**E14_W72**	Woman	72	Widow	Peddler	Manual	Primary school	Colon	3	No	Yes	No
**E16_W74**	Woman	74	Widow	Housewife	Manual	NA	Colon	2	No	No	No
**E17_W75**	Woman	75	Married	Secretary	Non manual	Secondary school	Rectum	3	No	Yes	Yes
**E18_W76**	Woman	76	Widow	Cook	Manual	Primary school	Colon	3	No	Yes	No
**E19_W78**	Woman	78	Widow	Housewife	Manual	Primary school (not finished)	Rectum	1	No	No	No

### Information gathering technique

The information was obtained through interviews. In accordance with the literature and the objectives of the study (relationship with the health system, experience of the process and coping strategies), we formulated a semi-structured interview guide which was tested and adapted during fieldwork. The interviewers were experienced interviewers (females) who had received specific training in qualitative methodology and interview techniques. After identifying potential participants, one of the researchers contacted general practitioners in order to confirm the patient’s eligibility. The same researcher phoned participants to schedule the interviews, which were conducted according to patient preferences (at private home or in their health centre), in the native language of the participants (Spanish or Catalan) and lasted approximately 1 h. The participants were provided with oral and written information about the study and they signed informed consent prior to the interview. In some cases relatives were present. The interviews were recorded in digital audio format and were transcribed verbatim. Transcripts were not returned to the participants.

### Analysis

Three researchers conducted a thematic content analysis [18]. Firstly, a code tree based on the general objectives of the study and the fieldwork data was created. This code tree was tested, agreed among analysts and modified in accordance with the new findings during analysis. The interviews were coded with the support of the Atlas.ti software. To verify the usefulness of the code tree and the validity of the process, two independent analysts double coded 8 interviews. After the inductive analysis procedures described above, codes were grouped into the categories proposed in the Lazarus y Folkman stress coping model [7, 19], a conceptual framework that has been used to understand stressful life events, adequate for our research as we consider the cancer diagnostic as a potential stressful factor. Table 2 provides an overall representation of the coping strategies obtained. Strategies are categorized as problem or emotion-centred, with emphasis on coping differences between the periods of illness and survival.

**Table 2:** Overall representation of coping strategies during illness and survival. Majorca 2017–2019

	**Problem-centred strategies**				**Emotion-centred strategies**	
		Confrontation	Acceptance	Distancing	Positive feelings	Negative feelings
**Illness**	Attitudes	Willpower, fighting spirit	Resignation and patience	Passivity, hope in health professionals Denial, self-sufficiency	Optimism, staying positive	Pessimism, sadness, anger…
	Actions	Understanding the disease	Daily routines (minimise impact)	No thinking Daily routines or new activities (escape-avoidance)		
**Survival**	Attitudes	Life-transforming changes Disorientation, feeling lost	Keep going	Forget Denial	Feeling proud Feeling lucky	Fear
	Actions	Lifestyles Psychotherapy, alternative therapies				

Social relationships are essential in coping with cancer [20], as well as other social determinants regardless of gender, age or cultural environment. However, due to its importance and breadth of this sub-theme, we have decided to address it in future work.

This study obtained the approval of the Majorca Primary Care Research Committee (nº PI11/12) and of the Balearic Islands Ethical Committee (IB3367).

## Results

Participants included 10 women and 11 men, the majority over 60 years of age, with half working in manual jobs. Many participants had only received education at primary school level. The disease (colon and rectal cancer) and its most common treatments (especially chemotherapy and colostomy) are homogeneously represented (Table 1).

The results are presented in two blocks: coping modes used during the disease and during survival. We divide each block into two sections: problem-centred and emotion-centred strategies (Table 2).

Pure coping strategies are not observed in this series. Instead, strategies are generally combined and even inconsistent. Furthermore, although coping patterns are sometimes identified, they vary within each person interviewed. Tables 3 and 4 show quotations selected based on clarity and relevance to illustrate the results.

**Table 3:** Quotations^a^ on problem- and emotion-centred strategies during illness. Majorca 2017–2019

**Strategies**	**Quotations**
**Attitudes**	
**Resignation**	**E07_W61**: I spent the night crying, all night, all night … but then I thought: “I am mortal, we are all mortal, if now it’s my time well … it’s my time.”
**Willpower, fighting spirit**	**E13_M71**: “I can, I can and I will” and I could and I did, that’s it (laughs). And that also helped me to assert myself, right? To say: “This is the way.”
**Passivity**	**E09_W67**: “[What did it mean for you the cancer diagnosis?] … Well, truth is that I barely took it in, I focused on getting it sorted, I trusted in the doctors and no, I didn’t even try.”
**Hope in health professionals**	**E01_W41**: [What strategies did you use to deal with it?] Strategies … when the doctors told me: “We are going to do this, it’s not pleasant”, “Now we are going to put a camera inside you”, I told them: “I just don’t care”. My phrase … from the moment I was diagnosed, anything they asked I said: “Whatever it takes.”
**Denial**	**E20_M80**: No, I’ve never felt [I have defeated cancer]. No, I’ve always felt I was okay, that what I had had was not real
**Self-sufficiency**	**E13_M71**: [what has helped me most has been] trusting my body, my strength, both physical and mental … right? My ability to analyse and face situations, that helped a lot
**Actions**	
**Daily routines**	**E20_M80**: [Did you go for coffee every day … even when you knew that you had cancer?] Yes, every day, every morning … It helped because I met with friends and was not locked up … I switched off, switched off
**No thinking**	**E11_M68**: I didn’t think about it too much, eh? I didn’t think about it too much because the more you think about it the worse, right? I continued to go to work and worked until shortly before my surgery
**Understanding the disease**	**E02_M46**: [What other things helped you cope with the disease during treatment?] Reading, I guess we all do it, I wanted information … of the disease, from prominent foreign oncologists, that explain the possible causes or origin of cancer to try and correct habits, to improve the situation
**Positive / negative feelings**	
**Optimism, staying positive**	**E09_W67**: It has a huge impact, some people are negative and other positive. And this has given me lots of strength, staying positive with all this
**Negativity (pessimism, sadness, anger…)**	**E12_W68**: Some people need to cry, some need to understand, some need to be angry, and some don’t quite understand

^a^The codes of the quotations refer to the interview number (ex. E01), followed by a letter that indicates the sex (W = woman, M = man) and a double digit that indicates the age (ex. M41)

**Table 4:** Quotations^a^ on problem- and emotion-centred strategies during survival. Majorca 2017–2019

**Strategies**	**Quotations**
**Attitudes**	
**Forget, keep going**	**E10_M68**: [Do you have any fears?] No, no, I say: “What will be will be” and I’m fine going [to the medical check-ups]. If they find something, then I’ll get knocked down again, but … I don’t go with the fear of: “What if they find something”, No. [A person who has defeated cancer has to resume their previous life, forget all about it.]
**Life-transforming changes**	**E02_M46**: Consequences of having survived cancer? What I just told you, a perspective on life a little … knowing to only sweat the really big stuff and this is good. This consequence is good, it teaches you to live better in some aspects
**Denial**	**E01_W41**: [How did you feel when they told you: “It’s over … you have defeated cancer”?] Well, I don’t know if there is a “it’s over” … even if they tell you: “it’s over”, you don’t quite believe it. I mean, I’ve been … for almost 9 years and you can’t quite believe that you’re healthy, that you’re fine. And you’re grateful and it doesn’t matter how you say it, but it’s like your mind can’t stop the fear of “what if …” and the “what if” is what prevails above all else
**Actions**	
**Lifestyles**	**E01_W41**: [What about physical activity or work?] Physical activity … for a while I even run, although I never saw myself as a runner. In addition, I have to say that I have taken supplements with lots of natural things, things that are now fashionable … And that I would recommend … reiki, all the combination of natural things with traditional medicine **E10_M68**: Well, before, I don’t know, you thought more about your life, to fight … and now you say: “What will be will be”, and if I want to eat something, then I eat it, and if I feel like doing something, I do. Before I used to over-think things
**Positive / negative feelings**	
**Feeling proud**	**E13_M71**: [Do you need to talk about cancer sometimes?] If I do it, it’s for self-esteem: “shhh! you don’t know who you’re talking to, I’ve survived the bug” (laughs) … I’m delighted, delighted, it has given me lots of confidence, yes, yes, yes. Not that I am immortal or invincible, but I’m strong, I’m strong, my body is ready and I can face almost anything
**Feeling lucky**	**E03_M54**: I feel lucky, really. In spite of everything, I feel very lucky because, I was just saying, I thought people died of cancer … there are still people, unfortunately, but not everyone, and I feel lucky
**Fear**	**E01_W41**: With all that happened this year I have to be super happy, but now I have my gyn check-up again … I live in constant fear, now I almost even laugh a little, but … I live scared, living scared after you know you don’t have it any more. But, my mind doesn’t want to know, it’s like my mind refuses to know that I’m okay

^a^The codes of the quotations refer to the interview number (ex. E01), followed by a letter that indicates the sex (W = woman, M = man) and a double digit that indicates the age (ex. M41)

### Coping during illness

The coping strategies during the disease originate from stressful experiences at the time of diagnosis or of treatments such as surgery or chemotherapy. Table 3 shows the quotations for this section.

#### Problem-centred coping strategies

We differentiate between three approaches when challenged with cancer: direct (confrontation); detached (distancing); and intermediate (acceptance). Strategies with a central cognitive component are categorised as attitudes and strategies with a central behavioural element as actions.

##### Attitudes

Regardless of age or gender, the most common strategy during illness is acceptance. Most participants explain that it is important to let time go by and to be patient because, eventually, they will adapt to the situation. They also frequently endorse confrontational positions such as willpower, strength and fighting spirit, which *they consider* beneficial to face these situations.

In contrast to this, some attitudes that involve detachment from the problem imply certain passivity in coping with the illness. Here, patients minimise their own role during these moments and stress the importance of letting professionals do their job. Placing hope in health professionals, generally in physicians, is another common theme that emerges when assessing what has helped in braving the disease.

Denial, i.e., refusing to acknowledge having experienced any type of distress, is considered an extreme example of distancing. Feedback with coping strategies close to self-sufficiency also emerged in the discourses, even a certain self-perception of invulnerability. It is essential to underscore reasoning’s that promote these attitudes, such as requiring a single surgery for the cancer or having suffered from other cancers. Extreme coping modes are not common, although men sometimes provided shorter answers aiming to belittle any suggestions of vulnerability. Women were more prone to explore these experiences and their coping strategies.

##### Actions

Coping can also involve a behavioural response represented through the confrontation-acceptance-distancing axis. Participants explained that leisure activities, work-related activities and daily routines helped coping with difficult moments of the disease. Keeping the usual routines and responsibilities after diagnosis facilitates adaptation. Leading a normal life, avoiding giving in to fear, getting out of the house and staying active were positively evaluated.

Concerning the need to continue the previous lifestyle or start new activities, participants stress the purpose for these actions closer to distancing: these activities allow us to disconnect, to avoid thinking and to put aside fears related to the illness and its possible consequences. Another common premise not linked to specific actions was avoiding obsessing over the problem.

Trying to understand the disease from a medical point of view constitutes another type of confrontation. In this case, patients acknowledge their need to read about cancer, its possible causes or how to prevent it. This coping mode has been observed in younger people; it is usually followed by doubts and might result in greater discomfort.

#### Emotion-centred coping strategies

Emotions are pivotal in the discourses of the people interviewed, who believe that they can play a favourable role in coping and communicate the need to control them. There is a prevailing belief that "you have to stay positive" when facing cancer, that it is important to think that everything will be alright and even to have (religious) faith. Numerous quotations convey the need for optimism and the positivity imperative with minimal variations.

The ubiquity of this type of quotation contrasts with the scarce reference to opposing discourses. The main examples of negative attitudes, generally considered unhelpful, include pessimism, sadness, desire to be alone, anger and feelings of unfairness, fragility and vulnerability. Perhaps to help ease the impact on them, participants sometimes explain that these feelings may be important and necessary to other people. None of the interviewees reported that expressing anger helped them cope with the disease, and there is advice on avoiding negativity. Notably, negativity predominantly appears in women’s discourses. Women described the need to openly express negativity as crying or being angry or not acceptance of their situation. They also mentioned the right of not fighting against the disease. A man mentioned that negativism is related with person’s personality.

### Coping during survival

Survival in cancer begins when the person is medically considered disease free with minimal chances of recurrence. The people interviewed do not talk so much about facing survival, a positive event that mitigates anxiety, but about how they cope with medical follow-ups, *sequelae* and, mainly, *uncertainty* and worry about a possible relapse. See Table 4 for quotations.

#### Problem-centred coping strategies

During survival we can also classify the results observed in attitudes and actions along the axis confrontation-acceptance-distancing.

##### Attitudes

Among the different ways of coping with uncertainty during survival, the most common attitude alternates between acceptance and distancing, forgetting and moving on, reiterating the need to turn the page, leaving the disease behind and not looking back.

As an example of confrontation, some people cope with survival through some life-transforming changes. They explain that cancer has changed them, that it has been an opportunity to see life anew, and that they now enjoy everyday things and appreciate what is really important. This experience of finding silver linings in having cancer is observed more often in younger people. Meanwhile they could still suffer from uncertainty of possible relapses and often feel lost or confused.

Most participants were overjoyed with the news of having defeated cancer and accepted that they were starting a new stage in their lives. We found some exceptions, indicating the extreme distancing of denial among participants who refused to acknowledge any impact of the disease and that survival is the beginning of a new phase. Another exception is found among the younger participants, who either because of their longer life expectancy they feel more at risk of a new cancer or for generational reasons, do not conclusively express joy or carelessness at having overcome the disease.

##### Actions

This section features daily routines and new activities with the purpose not only of mitigating survival anxiety, but also of confronting or distancing in the face of uncertainty. The relevance of lifestyles and, in younger participants, alternative therapies or psychotherapy, emerges at this stage.

Regarding helpful activities after surviving cancer, the most common coping strategy is improving lifestyles, mainly in relation to diet and physical activity. Advised by health professionals or found through self-education, participants might refer to these recommendations with frustration and even guilt due to compliance difficulties. Lack of compliance-willpower and the imprecision-multiplicity of recommendations were the main hindrances to success. The discomfort is sometimes explained as conflicting social imperatives, since daily obligations do not facilitate these behaviours.

Although many people interviewed talk about these activities and their preventive role regardless of their age, older interviewees do not show active interest in inquiring about these issues and minimise compliance.

#### Emotion-centred coping strategies

In illness and survival, positive feelings are considered helpful. Negative feelings are either deemed unhelpful or outright censored.

A positive feeling is feeling proud. Some patients underscore their ability to overcome adversity and their own worth as strengths that allow them to better face the concerns of this stage. This attitude is only observed in men. Feelings of gratitude are also common. Many participants acknowledge feeling lucky to have defeated the disease, since other patients have succumbed to it. On the other hand, feeling lucky is also perceived as an obligation, since not showing enough appreciation can be considered ungrateful.

The uncertainty that accompanies survival is rarely verbalized as fear; that is fear of relapse and of not managing to keep up with regular life, which is considered negative feelings to be avoided.

## Discussion

The results show differences in coping strategies during the stages of cancer diagnosis and treatment and survival. However, a common framework unites both stages: emotion and problem-centred strategies. With regard to the latter, during illness the most frequent coping strategies are acceptance and keeping daily routines, while denial and escape-avoidance behaviours are virtually absent. Here, interviewees particularly value willpower, a fighting spirit and avoiding thinking about the problem. During survival, acceptance and forgetting about the disease predominate, as well as activities aimed at improving lifestyles. Both strategies are considered very important once the cancer has been defeated. Regarding emotions, positive feelings, which are considered very useful, predominate in both stages. In contrast, negative feelings, generally considered unhelpful when facing cancer, are scarcely reported.

### Relationships between strategies and analysis of social conditioning

Coping models are not used in isolation and opposing attitudes coexist [21]. Paradoxical examples include participants who stress a fighting spirit and then explain that they resigned themselves, and men who emphasize their self-sufficiency when facing challenges, while explaining the care received from people around them. These contradictions encompass human vulnerability and interdependence as the universal framework of life experience, particularly during severe illness [22].

The links between different strategies point to coping patterns. For instance, during illness and survival some people use confrontational strategies, accessing their willpower and attempting to understand the disease. These interviewees describe survival by means of life-transforming changes, stressing lifestyle improvements. In contrast, other participants report more avoiding or accommodative strategies.

Sociocultural determinants such as gender, age and the social pressure of positive thinking might help explain certain coping mechanisms. Considering gender as a determinant of coping, women and men use similar strategies when facing cancer and the challenges of survival. However, only men’s discourses were associated with denial of discomfort, self-sufficiency, and pride in having defeated the disease and presenting themselves as *cancer experts* who help other sufferers. In contrast, while emphasizing positive feelings such as optimism, women tend to acknowledge and address negative feelings such as anger, wish to give up, need for solitude and the pain derived from loneliness. This is consistent with other studies, which indicate higher levels of anxiety in women, possibly because they express distress more freely than men [23]. These results show that the discourses reproduce gender stereotypes typical of patriarchal societies: male gender, associated with denial of interdependence and showing self-sufficiency; and female gender, in touch with their own emotions and open to acknowledge vulnerabilities and limitations [22]. In any case, it has been observed that both men and women could benefit from strong social networks [24].

Another finding is generational differences in coping. Older participants lean toward acceptance, the importance of daily routines and trying to keep going and improving lifestyles. Although only 7 interviewees were under 65 years of age, we identified common elements in their discourse, for instance more confrontational strategies, such as understanding the disease or finding a *good side* to cancer that has changed their perspective on life [25]. In this transformation, diet, exercise, psychotherapy and alternative therapies play a very important role. Younger people with cancer are more anxious and they develop less *effective* coping strategies than older people [26]. These differences might be explained by the severity of the disease, the treatment received or the longer life expectancy under the risk of a new cancer. We hypothesise that socio-historical conditioning factors (transition towards neoliberal economic models and their effects on work, lifestyles and subjectivity) might explain the differences in their discourses [27]. It would be necessary to investigate this aspect in future research.

Regarding emotion-centred strategies, we should underscore the scarce mention and low value attributed to negative feelings. In contrast, the insistence on staying positive is ubiquitous [6, 28, 29]. During survival, the attempts to feel lucky prevail, clouding negative feelings such as fear of relapse, which is not commonly directly addressed at this stage. Notably, the scientific literature also views this *positivity* favourably in relation to successful coping [30, 31]. However, censorship of negative feelings can be problematic, because they might promote adaptation to the new situation and seeking support from others. In addition, being overly optimistic and systematically dismissing the painful reality can be as harmful as negative feelings and pessimism. We consider more appropriate understanding that negative and positive feelings are contingent and dependent on the context. However, the literature hardly contains works that acknowledge the role of *negativity* [32]. Contesting the hegemony of positive thinking is crucial, since generally its historical and cultural conditioning factors are not taken into account. The encouragement of this type of *mindset* is relatively recent and probably stems from the business world of the 80 s [27] and then percolated into what is now known as *positive psychology* [33–35], recognizable in many of the referenced studies and interviewees’ discourses. This could partly explain the absence or rejection of negative feelings and the low presence of material determinants of coping, such as working conditions and financial situation. However, the participants did not systematically and passively reproduce this positive mindset, critical perspectives also emerged, as reported in relation to gender coping differences and when some participants rejected qualifying themselves as *survivors* [36, 37].

### Strengths, limitations and relevance

Some strengths of the study are: 1) We achieved a heterogeneous sample of participants obtained directly through the cancer registry, with specific inclusion criteria. 2) We carried out very open semi-structured interviews that were adapted to the settings, languages, agendas, and paces of the participants to facilitate their expression of emotions and narratives.

The interviewers were women, since it was thought that this would facilitate the participation and expression of the women interviewed. Regarding the men interviewed, it was assessed that they would have less resistance to elaborate their discourses with women interviewers, who also presented themselves as health workers [38].

It should be noted that the interviews were conducted with survivors, which naturally influences and transforms the discourse on coping with cancer. The *survivor bias* might cause the interviewee to overestimate certain strategies. On the other hand, this same limitation provides richness when exploring the experiences of cancer survivors, the main objective of this study.

Most participants of this study are older than 65 years, which could suggest that the results are only transferable to this age group. However, we have detected common patterns in younger participants, which indicates that saturation has been reached in the coping modes typical of these ages. Furthermore, we have formulated the hypothesis of a possible generational divergence.

Another limitation of this study is the selection of one type (colorectal) of cancer and the possibility that the results would only relate to experiences with this disease. However, the analysis has not focused on strategies that singularly address the particularities of this cancer and its treatment [39].

One could question the use of Lazarus y Folkman stress coping model, to establish the categories of analysis in this qualitative study. Although the categorization according to this model had not been initially planned, the results of the coding of the interviews showed its adequacy and coherence with our findings. This could be attributed to the fact that is a model used to understand stressful life events like cancer diagnosis.

## Conclusions

Although coping during illness and survival can be classified into common categories (problem and emotion-centred strategies), the challenges of these stages are faced differently. Age, gender and the cultural influence of positive psychology strongly influence both stages and strategies. These findings should guide further research and also encourage respectful and therapeutic treatment in all spaces of care of people who experience cancer treatment and survival.

## Data Availability

The datasets generated and/or analysed during the current study are not publicly available due individual privacy could be compromised but are available from the corresponding author on reasonable request.
